# Identification of diabetes self-management profiles in adults: A cluster analysis using selected self-reported outcomes

**DOI:** 10.1371/journal.pone.0245721

**Published:** 2021-01-22

**Authors:** Ketia Alexandre, Fanny Vallet, Isabelle Peytremann-Bridevaux, Olivier Desrichard

**Affiliations:** 1 School of Health Sciences (HESAV), University of Applied Sciences and Arts Western Switzerland (HES-SO), Lausanne, Switzerland; 2 Center for Primary Care and Public Health (Unisanté), University of Lausanne, Lausanne, Switzerland; 3 Faculté de Psychologie et des Sciences de l’Education, University of Geneva, Geneva, Switzerland; Fordham University, UNITED STATES

## Abstract

The present study describes adult diabetes self-management (DSM) profiles using self-reported outcomes associated with the engagement in diabetes care activities and psychological adjustment to the disease. We used self-reported data from a community-based cohort of adults with diabetes (N = 316) and conducted a cluster analysis of selected self-reported DSM outcomes (*i*.*e*., DSM behaviors, self-efficacy and perceived empowerment, diabetes distress and quality of life). We tested whether clusters differed according to sociodemographic, clinical, and care delivery processes variables. Cluster analysis revealed four distinct DSM profiles that combined high/low levels of engagement in diabetes care activities and good/poor psychological adjustment to the disease. The profiles were differently associated with the variables of perceived financial insecurity, taking insulin treatment, having depression, and the congruence of the care received with the Chronic Care Model. The results could help health professionals gain a better understanding of the different realities facing people living with diabetes, identify patients at risk of poor outcomes related to their DSM, and lead to the development of profile-specific DSM interventions.

## Introduction

Diabetes self-management (DSM) requires a proactive commitment to regularly and successfully performing multiple care activities in order to maintain proper control over the disease [1]. Many authors have highlighted the multifaceted nature of DSM [2–4]. Two dimensions of DSM are ubiquitous in the literature and constitute the basic structure of many DSM programs [5–7]. The first dimension involves diabetes care activities including decisions and actions about medication-taking (insulin or oral antidiabetic agents), being physically active, adjusting diet, self-monitoring of blood glucose (SMBG), and foot care [1, 8]. This dimension often encompasses constructs such as perceived diabetes self-efficacy and empowerment, as these are linked to an individual’s ability to complete diabetes care activities successfully. Perceived diabetes self-efficacy refers to the perceived ability and confidence to complete DSM tasks [9]. Perceived empowerment refers to involvement in goal-setting and decision-making [10]. The second dimension involves psychological adjustment and includes patients’ capacity to successfully adjust their lives to their illness via actions allowing them to control or reduce the disease’s impacts on their levels of physical, psychological, and social well-being [4]. Psychological adjustment encompasses constructs such as diabetes-related distress or quality of life (QoL) [4, 9].

Although DSM is associated with positive outcomes in terms of glycemic control, the prevention of complications, and improvements in QoL [11–14], daily DSM remains a challenge for most patients [15]. Indeed, previous studies have demonstrated that patients present different levels of disease self-management [15, 16]. The cross-national Diabetes Attitudes, Wishes and Needs study found that only 46% of type 1 diabetes patients (range across countries, 2%–63%) and 39% of type 2 diabetes patients (range across countries, 2%–54%) achieved complete success with at least two-thirds of their DSM activities [15]. According to recent systematic reviews by Coyle and colleagues, medication-taking remains a relatively frequent behavior among people with diabetes. However, variations existed regarding other DSM behaviors: daily rates of SMBG ranged from 42.4%–60%, modifications to diet ranged from 50%–80.9%, and daily foot care ranged between 17.4%–42.1%. Levels of physical activity were also found to vary across studies and not to change over time [16]. In addition, patients differed in terms of the barriers encountered and the resources available to them when performing their DSM activities [17, 18]. Indeed, the literature highlights that issues associated with DSM are multifactorial: psychological factors, as well as characteristics and skills associated with DSM behaviors, strongly influence DSM activities in adults. These types of influencing factors are obviously coherent with DSM’s two main dimensions of engagement in diabetes care activities and psychological adjustment [19].

To gain a better understanding of the issues surrounding DSM and to develop and evaluate DSM interventions, health policy institutions and professional organizations working in the field of diabetes have started to acknowledge and make good use of patient-centered, self-reported outcomes [9, 10, 20, 21]. The identification of patient profiles using selected self-reported outcomes from DSM’s two ubiquitous dimensions would help to explain patterns related to high/low levels of engagement in diabetes care activities and good/poor psychological adjustment to the disease. This information would broaden the scope of the interventions available to health professionals by taking into account the different realities and contexts of people’s DSM.

Surprisingly, the literature revealed a very limited number of studies specifically addressing the combined effects of related self-reported outcomes for defining adults’ DSM profiles [22–25]. These studies were limited to defining patterns within the population using either broadly defined variables [22] or, on the contrary, extremely specific psychosocial variables [23–25]. Moreover, these studies were performed on populations with specific characteristics (*e*.*g*., citizenship, diabetes type, duration of disease) or medical contexts (*e*.*g*., patients from a given diabetes clinic), but they never addressed a large, community-based cohort of type 1 and type 2 diabetes patients [22–25]. Research to inform the development of DSM interventions by identifying profiles specifically associated with selected self-reported DSM outcomes is sorely lacking. Besides, although previous studies looked at associations with sociodemographic and clinical characteristics, those related to care delivery processes were poorly addressed, such as recommendations for annual screening for late diabetes complications [22–25]. This was despite these issues being recognized as extremely important in the field of diabetes management [26, 27]. Another important aspect related to care delivery processes is congruence with the Chronic Care Model (CCM) [28, 29]. The CCM is an evidence-based conceptual framework describing the interacting system components (*i*.*e*., support to self-management, delivery system design, decisional support, and clinical information systems) that are important to providing quality care for chronic illness [28, 29].

The present study aimed to identify DSM profiles in a large community-based cohort of adults with diabetes. Specific study objectives were: 1) to describe distinctive DSM profiles by using selected self-reported outcomes in a cluster analysis using two dimensions—the dimension of engagement in diabetes care activities (DSM behaviors, empowerment, self-efficacy) and the dimension of psychological adjustment (diabetes distress, QoL), and 2) to describe these DSM profiles according to patients’ sociodemographic and clinical characteristics.

## Materials and methods

### Study design and population

This cross-sectional study used data from the 2014 follow-up of the CoDiab-VD cohort. This cohort was part of a longitudinal, population-based study monitoring the coverage and impact over time of a regional diabetes program on the quality of diabetes care in the canton of Vaud, Switzerland. Participants with either type 1 or type 2 diabetes were recruited in two stages (September 5 to October 15, 2011, and June 5 to July 15, 2012) through community pharmacies. Eligible participants were non-institutionalized adults (age ≥ 18 years) residing in the canton of Vaud and known to have had a diagnosis of diabetes for at least 12 months. Women with gestational diabetes, patients with suspected cognitive impairment as assessed by the pharmacist’s clinical judgment, and those unable to complete a written survey were not included [30]. CoDiab-VD participants were asked to complete a self-reported follow-up questionnaire sent to their home. More information regarding the CoDiab-VD cohort is registered with ClinicalTrials.gov (identifier NCT01902043), and its recruitment procedure has been published previously [31–33]. CoDiab-VD participants were representative of patients with diabetes in the canton of Vaud; their sociodemographic characteristics were similar to those of other surveys in Switzerland [33]. The Human Research Ethics Committee of the Canton of Vaud (Protocol N° 151/11) approved the study. Written informed consent was obtained from all cohort participants at the time of recruitment, and all data have been analyzed and kept anonymous and confidential.

### Measures

#### Variables used for the identification of DSM profiles

*Diabetes self-management behaviors*. DSM behaviors were measured using four of the six subscales of the validated French version of the extended Summary of Diabetes Self-Care Activities’ (SDSCA) questionnaire [34]. These DSM behavior subscales concerned: healthy eating, physical activity, SMBG, and foot care. Participants were asked to report the frequency with which they had performed these activities over the previous seven days, using an 8-point Likert scale ranging from 0 (never during the week) to 7 (every day of the week). For example: ‘‘How often did you follow your recommended diet over the last 7 days?”. Reliability analysis led to the elimination of two items: high consumption of fatty foods, from the healthy-eating subscale, and foot washing, from the foot-care subscale. After these eliminations, the four SDSCA subscales showed good reliability [healthy eating (four items): Cronbach’s alpha = 0.76; physical activity (two items): r = 0.51; SMBG (two items): r = 0.89; foot care (four items): Cronbach’s alpha = 0.62]; the four corresponding mean scores computed ranged from 0 (DSM behavior not performed at all) to 7 (DSM behavior performed every day) [34].

*Self-efficacy*. We assessed patients’ self-efficacy using the Diabetes Self-Efficacy questionnaire developed by the Sanford Patient Education Research Center [35]. This eight-item instrument assesses eight behavioral and medical management issues related to diabetes; it asks respondents to report their confidence in their ability to perform specific tasks (*e*.*g*., confidence in knowing what to do when their blood sugar level went higher or lower than it should be) using a 10-point Likert scale ranging from 1 (not at all confident) to 10 (totally confident). The eight items showed a good reliability coefficient (Cronbach’s alpha coefficient = 0.89). We computed the scale’s overall mean score, with higher scores indicating higher perceived self-efficacy [35].

*Empowerment*. We assessed patients’ feelings of empowerment brought on by DSM using the eight-item Diabetes Empowerment Scale–Short Form (DES–SF) [36]. This includes managing the psychosocial aspects of diabetes (4 items), assessing dissatisfaction and readiness to change (2 items), and setting and achieving goals (2 items). Participants were asked to report their degree of agreement using a 5-point Likert scale ranging from 1 (strongly disagree) to 5 (strongly agree). The eight items showed a good reliability coefficient (Cronbach’s alpha coefficient = 0.86). We computed the scale’s overall mean score, with higher scores indicating higher perceived empowerment [36].

*Diabetes distress*. We assessed patients’ emotional distress brought on by managing their disease using the Problem Areas In Diabetes (PAID-5) instrument [37]. Its five items cover a range of emotional states frequently reported in type 1 and type 2 diabetes: 1) being afraid when thinking about how to live with diabetes; 2) feeling depressed when thinking about how to live with diabetes; 3) being worried about the future and the possibility of serious complications; 4) feeling that diabetes requires too much mental and physical energy every day, and 5) being able to cope and respond to diabetes complications. Participants were asked to indicate their degree of emotional distress using a 5-point Likert scale ranging from 0 (not a problem) to 4 (a serious problem). The five items showed good reliability (Cronbach’s alpha coefficient = 0.94). Summing PAID-5’s responses gave a total score between 0 and 20, with a final score ≥ 8 indicating high distress [37].

*Quality of life (QoL)*. We assessed patients’ perceived QoL using the Audit of Diabetes-Dependent Quality of Life (ADDQoL) questionnaire [38]. Its 19 items assess the impact of diabetes in different areas of life: employment/career, social life, family relationships, friends, sex life, sport/leisure, own future, family’s future, motivation, physical activities, others fussing, and enjoyment of food. Participants were asked to rate each area of their life on how diabetes had affected its quality (from -3 “a great deal better”; -2 “quite a lot better”; -1 “a little better”; 0 “the same”; 1 “a little worse”; 2 “quite a lot worse”, and 3 “a great deal worse”) and the importance they attributed to that area (from 3 “very important”; 2 “important”; 1 “quite important”; and 0 “not at all important”). Each item had a “not applicable” option. Each item’s impact rating was multiplied by its corresponding importance rating to provide a score from -9 to +9. The 19 items showed good reliability (Cronbach’s alpha coefficient = 0.96). Weighted scores for applicable areas of life were first summed and then divided by the number of applicable areas to give a final score between -9 (maximum negative impact of diabetes) and +3 (maximum positive impact of diabetes) [38].

#### Variables used for the comparison of DSM profiles

*Sociodemographic variables*. Sociodemographic variables included age, sex, employment status, feelings of financial insecurity measured by the difficulty in paying household bills over the last 12 months (yes or no), and the number of individuals living in the household.

*Clinical variables*. These variables included diabetes type (I or 2), treatment type (oral antidiabetic or/and insulin), body mass index (BMI, self-reported weight (kg)/height (cm)^2^), smoking status (yes or no), health literacy [measured as having experienced difficulty understanding written information about medical treatment or health status (yes or no)] [39, 40], and patients’ knowledge of their HbA1c and blood pressure values (yes or no). In addition, we used patients’ comorbidity score, as measured by the number of reported comorbid conditions (*e*.*g*., heart failure, cancer), and depression screening score, measured using the Two-Question Screen [41] for the detection of depression.

*Care delivery process variables*. These variables included recommended annual screening for late-stage diabetes complications and the congruence of the care received with that of the CCM. We assessed the reception of recommended annual screening for late-stage diabetes complications using patients’ responses regarding the completion of the following recommended checks, in the past twelve months, by a health professional (yes or no): HbA1C levels, blood pressure measurement, lipid profile, diabetic foot examination, urine test for microalbuminuria, and eye examination by an ophthalmologist. We assessed the congruence of the care received with that of the CCM by using the Patient Assessment of Chronic Illness Care (PACIC) questionnaire [42]. The PACIC assesses the congruence of the chronic care received with that of the CCM by examining patients’ evaluation of care received for their own chronic illness. The questionnaire includes 20 items measuring the extent to which patients report receiving care congruent with that of the CCM in the following domains: patient activation, delivery system design, goal-setting, problem-solving, and follow-up/coordination over the past six months (5-point Likert scale, ranging from 1 “almost never” to 5 “almost always”). The 20 items showed good reliability (Cronbach’s alpha coefficient = 0.94). We computed the scale’s overall mean score as suggested in a recent work by Iglesias and colleagues, with higher scores indicating greater involvement in self-management and the receipt of chronic care counselling by health professionals [43].

### Statistical analysis

First, we performed descriptive univariate analyses (*i*.*e*., frequencies, percentages, mean scores, and standard deviations). Successively, we converted the scores of variables used for cluster identification into standardized values since each instrument had different scales and units. These statistical procedures were applied to the average value of the diabetes distress variable (PAID-5, range 0–4), which was then rescaled, for reporting purposes, to a range from 0–20 to simplify comparisons with previous literature [37]. We followed Clatworthy and colleagues’ procedure for cluster identification and validation [44]. To determine the number of clusters, we visually examined the dendrogram (agglomeration schedule) and looked for sudden, large increases when measuring similarities between joined clusters. We then compared two classification methods to test the robustness of the clusters identified: the agglomerative hierarchical procedure, using Ward’s method, and the iterative partitioning method, using k-means. We calculated Cohen’s kappa coefficient to measure the level of agreement between the distributions of participants in the clusters identified by both methods. Finally, we tested whether the clusters differed according to sociodemographic, clinical care, and care delivery process variables using one-way ANOVA for continuous variables and χ^2^ comparison for categorical variables. All analyses were conducted with SPSS statistical software, version 23.0.

## Results

### Participants’ characteristics

Of 519 CoDiab-VD cohort participants recruited in 2011–2012, we sent the 2014 follow-up questionnaire to the 402 participants not lost to follow-up, and 339 completed and returned it (84.3% response rate). Among these responders, 316 had no missing self-reported data on the variables used for DSM profile identification, and they became our sample group. The sample’s average age was 66 years old, it was mainly composed of men, 28% stated that they were professionally active, and approximately 23% reported living alone. Nineteen percent reported that paying household bills had been a problem over the last 12 months. Most participants reported having type 2 diabetes, and about half had had this diagnosis for at least ten years and were taking oral antidiabetic treatment. The sample’s average BMI was 30 kg/cm^2^, and more than half reported two or more comorbidities, with approximately one-third scoring positively on the depression-screening test (Table 1).

**Table 1:** Patients’ sociodemographic and clinical characteristics (N = 316).

Variables (available number of participants)	N (%) or *M* ± SD	Min–Max value
Age	65.6 ± 0.4	28–89
Sex		
Female	119 (37.7)	
Male	197 (62.3)	
Professionally active (N = 296)	89 (28.2)	
Living alone (N = 315)	74 (23.4)	
Had difficulties paying household bills over the last 12 months (yes) (N = 308)	60 (19.0)	
Type of diabetes (N = 308)		
Type 1	43 (13.6)	
Type 2	265 (83.9)	
Type of treatment		
Oral antidiabetic (yes) (N = 314)	233 (73.7)	
Insulin (yes) (N = 314)	166 (52.5)	
Comorbidity scoring (1 to 7) (N = 313)		
None	61 (19.3)	
1	84 (26.6)	
2	81 (25.6)	
3	53 (16.8)	
≥ 4	34 (10.8)	
Positive depression-screening (N = 314)	96 (30.4)	
Smoking status (current) (N = 309)	57 (18.0)	
BMI kg/cm2 (N = 305)	30.1 ± 5.6	18.0–51.7

BMI, body mass index.

### Identification of DSM profiles

**Fig 1:**
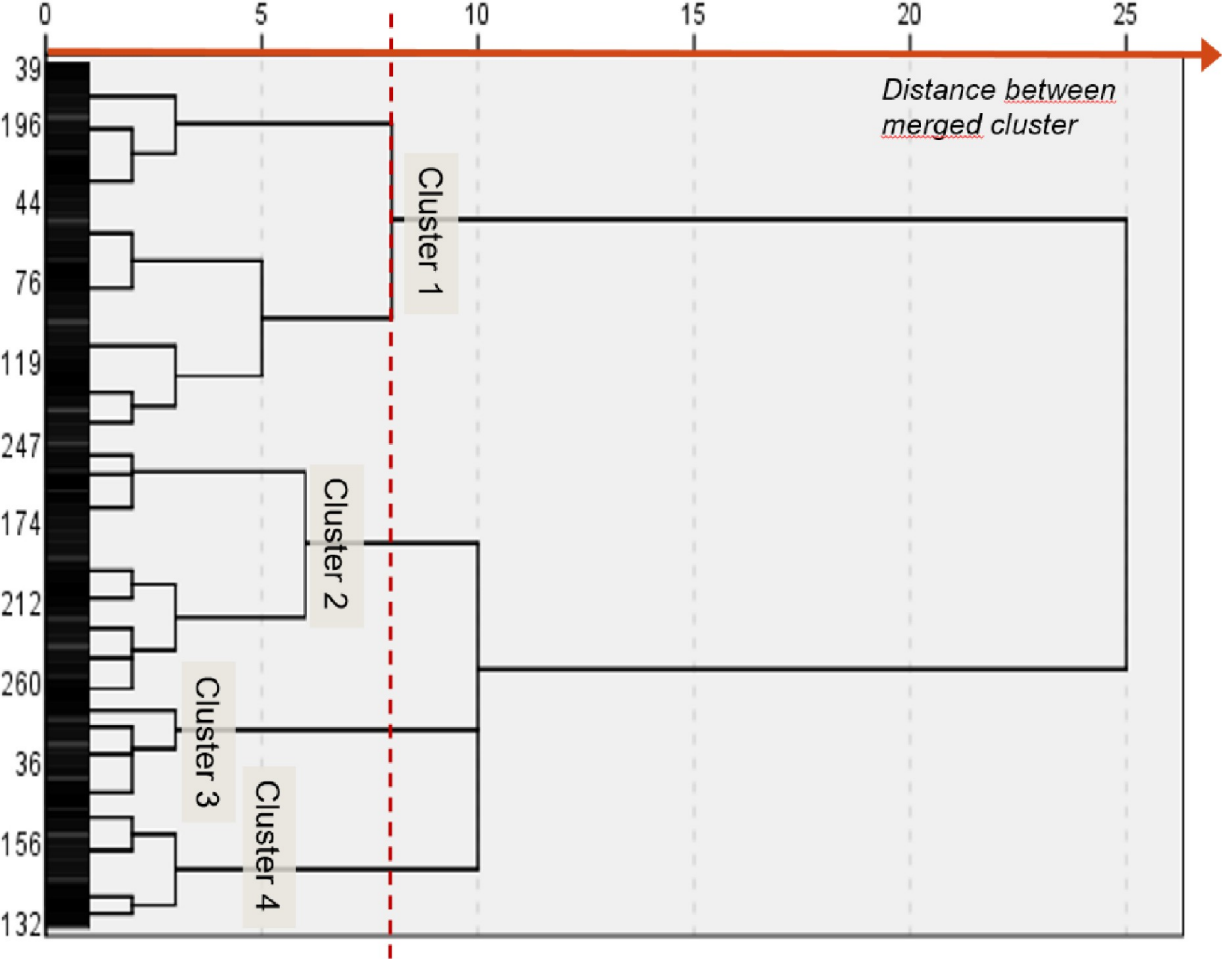
Dendrogram diagram for cluster identification. The distance between the merged clusters indicates the optimal cluster solution for four clusters.

**Fig 2:**
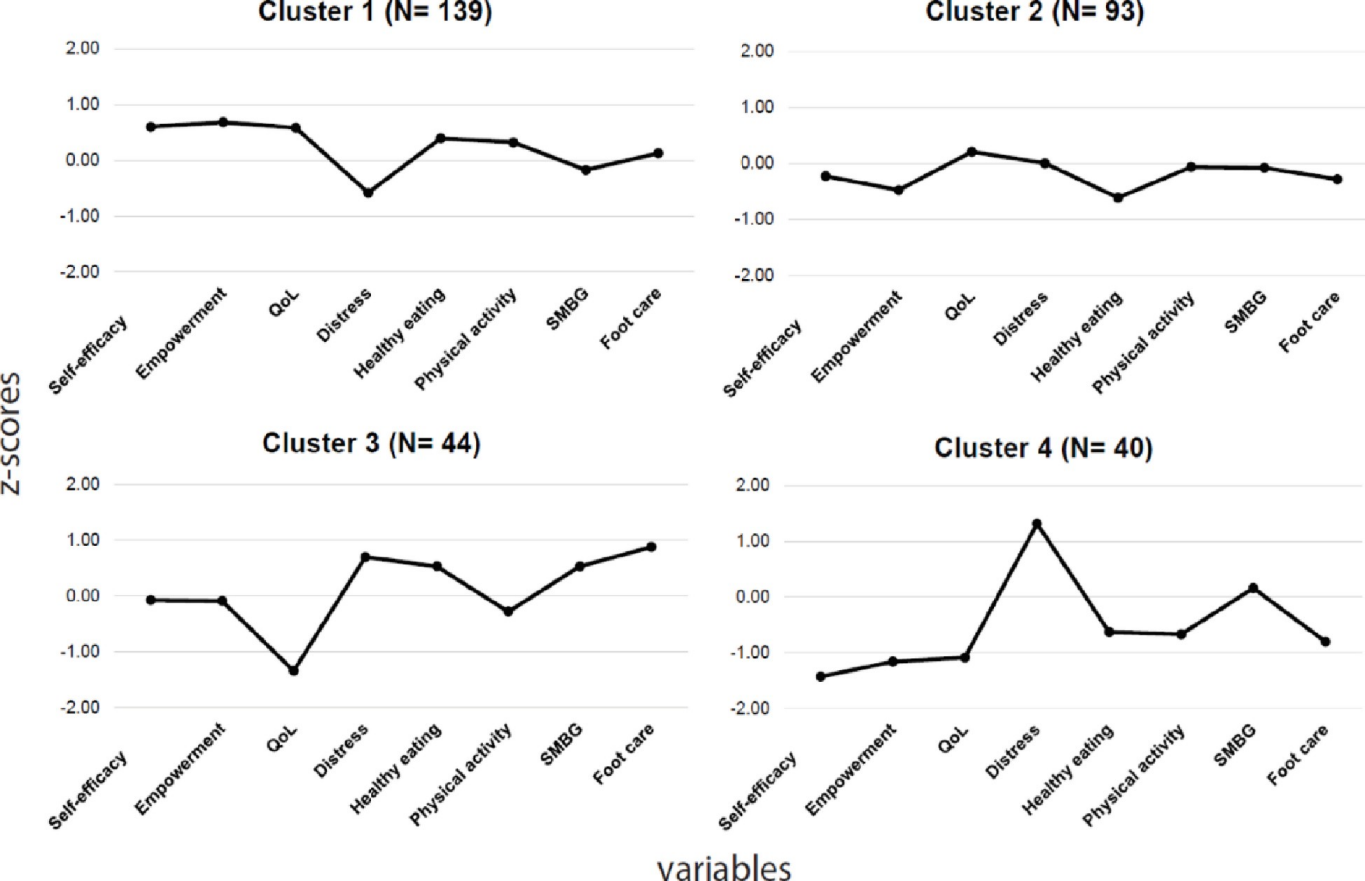
Standardized scores for each variable used for cluster identification. QoL, quality of life; SMBG, self-monitoring of blood glucose; the z-score of diabetes distress variable was not inverted.

An initial visual examination of the dendrogram identified four final clusters; an additional cluster merging with them would have implied a major drop in their internal homogeneity (Fig 1). This choice represented the best compromise between intra-group homogeneity and the number of independent clusters to analyze. Fig 2 presents the z-scores of the eight variables used for cluster identification: for each scale, high z-scores indicated a higher perception or performance of the variable. A k-means analysis confirmed that the sample’s optimal number of clusters was four, with profiles very close to those obtained using hierarchical methods. The number of participants in the k-mean clusters differed only slightly from those identified using the hierarchical method. The κ coefficient of 0.57 indicated moderate agreement between the two clustering methods [45], confirming that the clusters had been identified in a similar manner.

Table 2 presents the mean scores for each profile on each variable and the results of Tukey *post-hoc* comparisons. The results indicated that the four clusters defined distinct patient DSM profiles and showed significant differences in terms of the eight variables related to DSM. Profiles were also quite distinct for the empowerment and distress variables. In addition, our classification was not merely the result of simple linear relationships between the variables: the four clusters displayed different combinations of characteristics (Table 2).

**Table 2:** Mean scores for each of the psychosocial variables used for the identification of DSM profiles.

	CoDiab-VD 2014 participants (N = 316)	Profile 1 High self-appraisal (n = 139)	Profile 2 Limited-engagement (n = 93)	Profile 3 Strained (n = 44)	Profile 4 Distressed (n = 40)	
Variables (Instrument/scale range low to high)			*M *± SD			F
Self-efficacy	7.9	8.7^a^	7.5^b^	7.8^b^	5.4^c^	87.3*
*Stanford 8 items/1 to 10*	1.7	1.0	1.4	1.5	1.7	
Empowerment	4.1	4.5^a^	3.7^b^	4.0^c^	3.2^d^	84.2*
*DES-SF 8 items/1 to 5*	0.7	0.4	0.6	0.6	0.7	
Diabetes distress	5.3	2.4^a^	5.3^b^	8.5^c^	11.8^d^	81.3*
*PAID 5 items/0 to 20*	5.0	3.1	3.8	4.9	3.9	
Quality of life	-1.5	-0.5^a^	-1.1^b^	-3.7^c^	-3.3^c^	134.2*
*ADDQoL 19 items/-9 to +3*	1.7	0.6	1.1	1.5	1.8	
Healthy eating	4.8	5.4^a^	3.9^b^	5.6^a^	3.9^b^	39.2*
*SDSCA 4 items/0 to7*	1.4	1.2	1.4	1.0	1.3	
Physical activity	2.9	3.6^a^	2.8^ab^	2.3^bc^	1.5^c^	13.3*
*SDSCA 2 items/0 to 7*	2.1	2.6	1.8	2.2	1.2	
SMBG	4.2	3.7^a^	4.0^a^	5.7^b^	4.7^ab^	6.3*
*SDSCA 2 items/0 to 7*	2.7	2.8	2.8	2.0	2.5	
Foot care	3.2	3.5^a^	2.8^a^	4.8^b^	1.8^c^	28.8*
*SDSCA 4 items/ 0 to 7*	1.8	1.8	1.4	1.2	1.2	

When averages do not share the same letter, they differ significantly in Tukey *post-hoc* comparisons

* *p* < 0.000; DES-SF, Empowerment Scale–Short Form; PAID, Problem Areas In Diabetes; ADDQoL, Audit of Diabetes-Dependent Quality of life; SDSCA, Summary of Diabetes Self-Care Activities; SMBG, self-monitoring of blood glucose.

**Fig 3:**
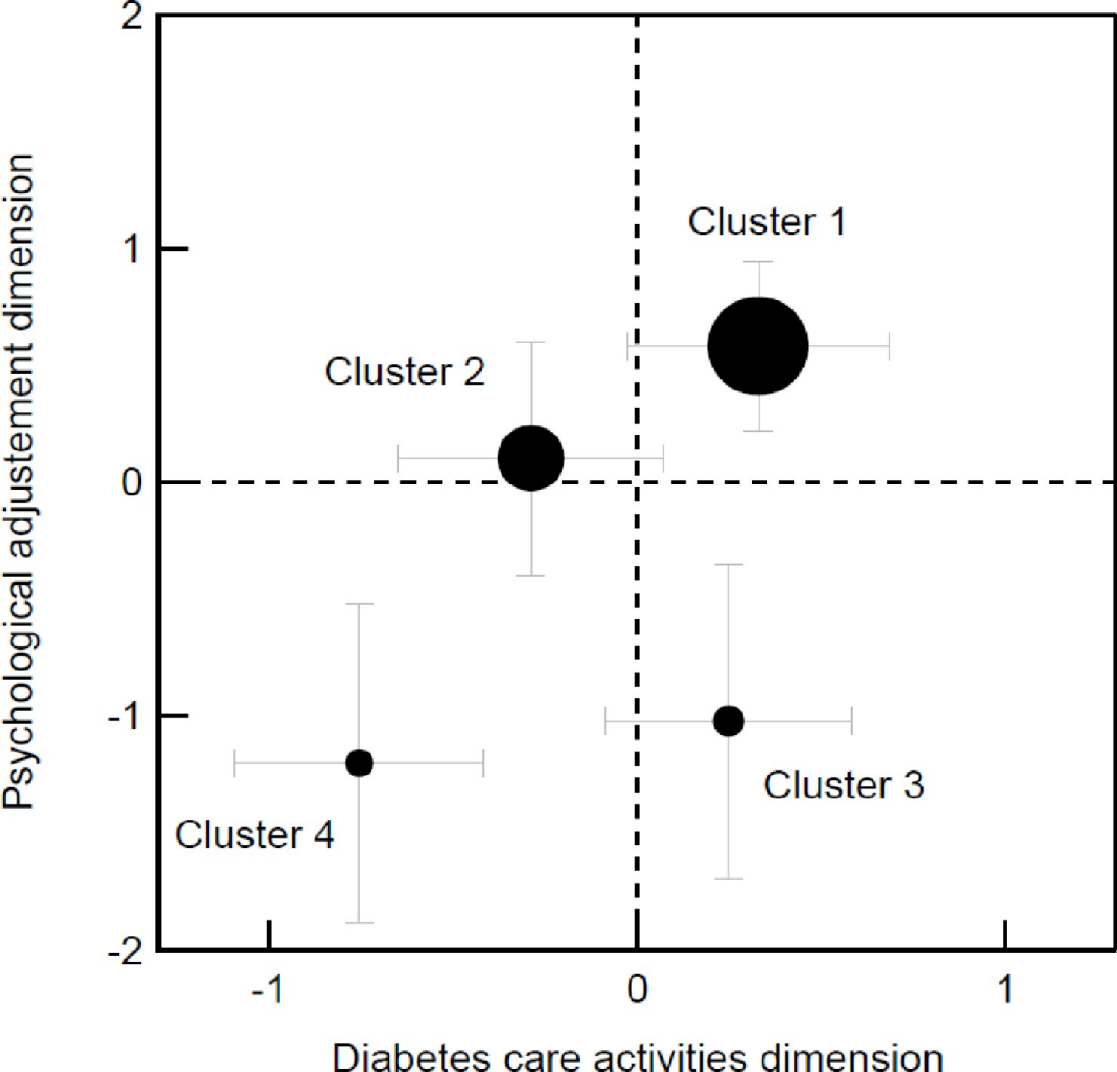
2D Cartesian plot obtained using the averaged z-scores from the variables used to identify clusters. (a) Variables related to the diabetes care activities dimension (DSM behaviors, self-efficacy, and perceived empowerment) featuring along the horizontal axis; (b) Variables related to the psychological adjustment dimension (diabetes distress and perceived quality of life) feature along the vertical axis; The distress variable’s z-score was inverted; The disk diameter is proportional to the cluster size: total number of participants N = 316, cluster 1 n = 139, cluster 2 n = 93, cluster 3 n = 44, clusters 4 n = 40; The error bars indicate each cluster’s standard deviation.

The four clusters obtained can be visualized on a 2D Cartesian plot by setting each cluster’s position along the axes using the averaged z scores from the variables used for cluster identification (Fig 3). Variables from the diabetes care activities dimension (DSM behaviors, self-efficacy, and perceived empowerment) defined the horizontal axis, and variables from the psychological adjustment dimension (diabetes distress and QoL perceived) defined the vertical axis. The direction of the z-score for the diabetes distress variable was inverted before averaging for consistency with the other variables’ definitions.

### Other results from the comparative analysis of DSM profiles

The results of cluster comparisons using sociodemographic, processes of care, and clinical variables are reported in Table 3. One-way ANOVA using χ2 analyses found no significant differences between profiles for age, sex, employment status, living alone, type of diabetes, smoking status, level of health literacy, knowledge about blood pressure, or the completion of an annual urine check. The variables of knowledge about HbA1c, and completed annual check-ups for lipids, blood pressure, eyes examination, and HbA1c could not be accounted for in this comparison because the numbers of answers collected for certain clusters were too small to allow a reliable χ^2^ determination. The analyses indicated significant χ^2^ coefficients for insulin treatment, comorbidity score, depression screening, and financial insecurity. One-way ANOVA tests for continuous variables found a significant F statistic for BMI and variables of patients’ assessment of the chronic care received (Table 3).

**Table 3:** DSM profile comparison according to patients’ sociodemographic, processes of care, and clinical variables.

	CoDiab-VD 2014 participants (N = 316)	Profile 1 High self-appraisal (n = 139)	Profile 2 Limited-engagement (n = 93)	Profile 3 Strained (n = 44)	Profile 4 Distressed (n = 40)	
***Sociodemographic variables***						
Age						
*M*	65.6	66.8	65.1	65.7	62.9	F = 1.6
*SD*	10.4	9.9	10.3	11.4	11.0	
Sex						
Male						
n	197	88	59	22	28	χ^2^ = 4.0
%	62.3	63.3	63.4	50.0	70.0	
Professionally active						
n	89	38	30	6	15	χ^2^ = 6.4
%	28.2	28.6	34.1	15.8	40.5	
	(N = 296)	(n = 133)	(n = 88)	(n = 38)	(n = 37)	
Living alone						
n	74	33	23	8	10	χ^2^ = 0.9
%	23.4	23.7	25.0	18.2	25.0	
	(N = 315)		(n = 92)			
Had difficulties paying household bills over the last 12 months						
n	60	17	13	14	16	χ^2^ = 22.2**
%	19.0	12.5	14.3	34.1	40.0	
	(N = 308)	(n = 136)	(n = 91)	(n = 41)		
***Clinical variables***						
Type of diabetes (Type 2)						
n	265	117	83	34	31	χ^2^ = 2.4
%	83.9	86.7	89.2	81.0	81.6	
	(N = 308)	(n = 135)		(n = 42)	(n = 38)	
Taking insulin treatment						
n	166	57	49	31	29	χ^2^ = 19.5**
%	52.5	41.0	53.8	70.5	72.5	
	(N = 314)		(n = 91)			
Comorbidity scoring ≥2						
n	168	61	54	29	24	χ^2^ = 9.4*
%	53.2	44.2	59.3	65.9	60.0	
	(N = 313)	(n = 138)	(n = 91)			
Positive depression screening						
n	96	19	27	20	30	χ^2^ = 63.4**
%	30.4	13.7	29.0	46.5	76.9	
	(N = 314)			(n = 43)	(n = 39)	
BMI^†^						
*M*	30.1	29.2^a^	30.0^ab^	31.9^b^	31.4^b^	F = 3.2*
*SD*	5.6	5.4	5.3	5.7	6.3	
	(N = 305)	(n = 135)		(n = 41)	(n = 36)	
Currently smoking						
n	57	23	18	6	10	χ^2^ = 2.0
%	18.0	17.3	19.6	13.6	25.0	
	(N = 309)	(n = 133)	(n = 92)			
Health literacy ability: experienced difficulty understanding written information about medical treatment or health status (yes)						
n	99	34	31	17	17	χ^2^ = 7.3
%	31.3	24.8	33.7	39.5	44.7	
	(N = 310)	(n = 137)	(n = 92)	(n = 43)	(n = 38)	
***Care delivery process variables***						
Patients’ assessment of chronic care model received-PACIC^†^ (scale: 1 lowest to 5 highest)						
*M*	2.7	2.7^ab^	2.6^b^	3.0^a^	2.6^b^	F = 2.9*
*SD*	0.9	1.0	0.9	0.8	0.8	
	(N = 311)	(n = 137)	(n = 90)			
Knowledge about HbA1C (yes)^††^						
n	253	115	70	35	33	
%	80.1	87.8	87.5	83.3	89.2	
	(N = 290)	(n = 131)	(n = 80)	(n = 42)	(n = 37)	
Knowledge about blood pressure (yes)						
n	198	81	65	29	23	χ^2^ = 3.5
%	62.7	61.8	73.9	67.4	63.9	
	(N = 298)	(n = 131)	(n = 88)	(n = 43)	(n = 36)	
Annual HbA1C check completed (yes)^††^						
n	273	124	74	40	35	
%	86.4	93.2	88.1	95.2	97.2	
	(N = 295)	(n = 133)	(n = 84)	(n = 42)	(n = 36)	
Annual lipid check completed (yes)^††^						
n	291	127	87	42	35	
%	92.1	94.8	97.8	97.7	94.6	
	(N = 303)	(n = 134)	(n = 89)	(n = 43)	(n = 37)	
Annual eye check completed (yes)^††^						
n	290	126	86	43	35	
%	95.4	93.3	95.6	100.0	97.2	
	(N = 304)	(n = 135)	(n = 90)	(n = 43)	(n = 36)	
Annual urine check completed (yes)						
n	209	90	63	37	19	χ^2^ = 7.5
%	66.1	71.4	78.8	88.1	63.3	
	(N = 278)	(n = 126)	(n = 80)	(n = 42)	(n = 30)	
Diabetic foot examination completed (yes)						
n	190	85	49	33	23	χ^2^ = 6.0
%	60.1	65.9	54.4	75.0	63.9	
	(N = 299)	(n = 129)	(n = 90)		(n = 36)	
Blood pressure measurement completed (yes)^††^						
n	304	133	91	43	37	
%	96.2	97.8	98.9	100.0	97.4	
	(N = 309)	(n = 136)	(n = 92)	(n = 43)	(n = 38)	

Each time the number of individuals in a specific profile differs from the total CoDiab-VD sample or the total number in the profile, the actual sample size is reported in parentheses; Tests applied: F comparison for continuous variables, χ^2^ comparison for categorical variables; *Post-hoc* comparisons are presented when statistical analysis allowed them to be calculated; † When averages do not share the same letter, they differ significantly in Tukey *post-hoc* comparisons; †† The number of answers for some profiles was too small to calculate a reliable χ^2^ value for comparison

* *p*< 0.05

** *p* = 0.000; BMI, body mass index.

### Description of the four DSM profiles

The following section presents narrative descriptions of the four DSM profiles.

#### Cluster 1: High self-appraisal profile (n = 139, 44.0%)

In the top-right quadrant (Fig 3), this profile shows a good balance between the performance of self-reported care activities and psychological adjustment to the disease. This profile had the highest scores for self-efficacy, empowerment, and QoL, and the lowest scores for distress. Moreover, on average, high self-appraisers had positive scores for all DSM behaviors except SMBG, which was the least frequent behavior. With respect to other profiles, high self-appraisal patients showed differential sociodemographic and clinical variables: low rates of financial difficulties, insulin treatment, comorbidities, and depression. They also displayed lower BMIs than strained and distressed profiles.

#### Cluster 2: Limited-engagement profile (n = 93, 29.4%)

In the top-left quadrant (Fig 3), we identified a profile of patients with low rates of self-reported DSM but midrange scores for psychological adjustment, with no notably weak or strong variables. Patients’ scores for DSM behaviors, self-efficacy, and perceived empowerment were lower than those of high self-appraisal and strained profiles. Moreover, these patients seemed less affected by the disease and distress related to disease management, and these had less impact on their QoL than they did for strained profile patients. Considering sociodemographic and clinical variables, limited engagement patients were somewhere between the high self-appraisal and strained profiles when it came to taking insulin treatment and depression. They differed from strained and distressed profiles when it came to financial difficulties and from high self-appraisers on comorbidities.

#### Cluster 3: Strained profile (n = 44, 13.9%)

Contrary to limited engagement profile patients, strained profile patients (bottom-right quadrant, Fig 3) were characterized by average rates of self-reported DSM behaviors, good self-efficacy and perceived empowerment, high diabetes distress, and poor QoL. This profile highlights the gap between good diabetes care and poor psychological adjustment to the disease. High scores for distress contrasted with low scores for QoL. This profile’s QoL score was the lowest, but patients reported higher levels of self-efficacy and empowerment than the limited engagement and distressed profiles. Patients in the strained profile were the least physically active but reported the highest rates of SMBG. Strained patients had higher scores than high self-appraisal and limited engagement patients for financial difficulties, taking insulin treatment, comorbidities, depression, and BMI.

#### Cluster 4: Distressed profile (n = 40, 12.7%)

This profile (bottom-left quadrant, Fig 3) combined individuals with poor diabetes self-management, with the lowest average rates of DSM behaviors and of psychological adjustment. Compared to the other profiles, patients in the distressed profile reported the highest distress and the lowest self-efficacy and empowerment. They also presented with the lowest rates of DSM behaviors, except for SMBG, which was, on average, comparable to the high self-appraisal and limited engagement profiles. Distressed patients shared similar clinical variables scores with patients from the strained cluster, but had by far the highest proportion of patients (76.9%) with a positive depression-screening score.

## Discussion

This study identified DSM profiles in a large community cohort of adults with diabetes. Using selected self-reported outcomes from two dimensions of DSM—diabetes care activities (*i*.*e*., DSM behaviors, empowerment, and self-efficacy) and psychological adjustment to the disease (*i*.*e*., diabetes distress and quality of life)—cluster analysis revealed four distinctive DSM profiles. The four profiles (high self-appraisal, limited engagement, strained, and distressed) combine high/low levels of engagement in diabetes care activities and good/poor psychological adjustment to the disease. Although self-reported DSM outcomes were good in the high self-appraisal profile cluster and poor in the distressed profile cluster, our results indicated that in the limited engagement and strained profile clusters, the self-reported capability to efficiently perform diabetes care activities did not automatically imply psychological well-being, and *vice versa*. In addition, the profiles are characterized by variables relating to perceived financial insecurity, taking insulin treatment, having depression, and the care received’s congruence with that of the CCM. These results should help health professionals gain a better understanding of the DSM experience among adults with diabetes, identify patients at risk of poor outcomes related to DSM, and develop more specific follow-up interventions based on the DSM profiles identified.

One important strength of our study was its identification of each profile’s specific needs for their daily DSM processes. Although several systematic reviews have reported successful interventions for supporting DSM in general [11–14], there is still a lack of interventions specifically tailored to distinct DSM profiles. The profiles identified in the present work were independently validated using a set of sociodemographic and clinical variables congruent with the literature on DSM [2, 20, 21], and they will help the development of profile-specific intervention targets. Although the high self-appraisal, limited engagement, and distressed profiles share many similarities with profiles proposed in previous studies [22–25], our results highlighted the existence of a strained profile representing a significant subpopulation (13%) that health professionals should be vigilant for. In addition, this study contributes to informing researchers about the best direction for and the degree of importance of interventions to support DSM for each profile. This is because it relies on selected self-reported outcomes from the two ubiquitous dimensions of DSM that are disease management and psychological adjustment. Keeping in mind that there are several different profiles of DSM should help to increase the accuracy and efficiency of future interventions.

Our results found no associations between variables relating to different recommended types of annual screening and a patient’s specific profile. This observation had been acknowledged previously by other authors [23]. Our results also revealed that patients with profiles characterized by high rates of diabetes self-care activities (high self-appraisal and strained profiles) received care congruent with that of the CCM. Our results highlighted both that disease management could be successful despite poor psychological adjustment and that good psychological adjustment does not strictly imply a higher rate of diabetes care activities. Therefore, continuous efforts should be made to implement DSM promotion interventions that fully consider the dimensions of disease management and psychological adjustment.

Our results should be discussed in light of several limitations. First, the kappa index obtained from the cross-validation of two clustering methods is in the middle range. Although this validation step is recommended in cluster analysis, the expected outcomes are not clearly defined. We considered the value of 0.57 to be acceptable for the confirmation of the profiles. Second, although the variables used for DSM profiling are broadly recognized self-reported DSM outcomes, other variables (*e*.*g*., personality type, beliefs about DSM behaviors, access to care, and the social support received from family or peers) were neglected and may need to be considered in future studies. Likewise, a study design using less subjective indicators of self-management (*e*.*g*., HbA1C levels) would be useful for better defining DSM profiles. Third, the differences observed when comparing profiles against sociodemographic, processes of care, and clinical variables came from bivariate analyses, and they did not consider a possible adjustment model of the variables between themselves. Fourth, the study population’s average age was higher than that observed in other studies. Indeed, further studies should target different age groups to determine age’s impact on different DSM profiles. Finally, our results came from self-reported data in response to pre-defined questions; the inclusion of a qualitative component could complement these results and lead to an even better understanding of the different profiles.

### Implications for practice

The present study’s results provide information of interest to health professionals facing the many dimensions and significant variability of everyday DSM among adults. Interventions aimed at promoting DSM among patients might benefit from the identification of different DSM profiles. Patients’ feedback on their personal experiences could help determine the most appropriate profile. Following the identification of typical DSM profiles, interventions can be designed according to specifically targeted dimensions of DSM. Profile-specific interventions should include: 1) valuing and supporting the long-term maintenance of the equilibrium between disease management and psychological adjustment among the high self-appraisal profile cluster; 2) strengthening the motivation for DSM among the limited-engagement profile cluster; 3) helping to manage disease-related worries and make life-style adjustments to the disease among the strained profile cluster and; 4) taking a variety of robust actions (*e*.*g*., treatment of depression and addressing issues related to comorbidities, insulin treatment, and the negative effects of financial insecurity) among the distressed profile cluster. Finally, the profile names should be replaced by generic labels (*e*.*g*., colors) to avoid patients developing any feelings of being judged [46].
